# The Influence of Terfenol-D Content on the Structure and Properties of Multiferroic Composites Obtained Based on PZT-Type Material and Terfenol-D

**DOI:** 10.3390/ma18020235

**Published:** 2025-01-08

**Authors:** Dariusz Bochenek, Artur Chrobak, Grzegorz Dercz, Przemysław Niemiec, Dagmara Brzezińska, Piotr Czaja

**Affiliations:** 1 Institute of Materials Engineering, Faculty of Science and Technology, University of Silesia in Katowice, 75 Pułku Piechoty 1a, 41-500 Chorzów, Poland; artur.chrobak@us.edu.pl (A.C.); grzegorz.dercz@us.edu.pl (G.D.); 2 Institute of Technology, University of the National Education Commission, Podchorążych 2, 30-084 Kraków, Poland; piotr.czaja@up.krakow.pl

**Keywords:** Terfenol-D, PZT, multiferroic composites, dielectric properties, magnetic properties

## Abstract

In this work, three composite materials based on Terfenol-D and PZT-type material were obtained with a classic sintering method using a combination of 0–3 phases, where the ferroelectric phase was doped PZT material (P) and the magnetic phase was Terfenol-D (T). The percentage of P and T components in the composites was variable, i.e., 90% P/10% T (P90-T10), 70% P/30% T (P70-T30), and 50% P/50% T (P50-T50). Structural, microstructure, dielectric, and magnetic properties and DC electric conductivity of multiferroic composites were investigated. Chemical composition analyses and X-ray studies showed a decomposition of the composite compositions, forming additional phases, most of which contained rare earth elements and Fe. Microstructural SEM-BE (backscattering) images distinguished areas of bright intensity with a dominant ferroelectric phase and dark areas with a dominant magnetic element dominance. Despite the composition decomposition, the composite materials retained good dielectric and magnetic properties at room temperature. The highest stability of dielectric parameters was maintained by the P90-T10 composition with high values of permittivity *ε* = 570 at room temperature RT (*ε*_m_ = 7300 at the phase transition temperature *T*_m_) and the lowest dielectric tangent loss (tan*δ* of 0.32 and 1.94 for RT and *T*_m_, respectively). Increasing the Terfenol-D share in the composite causes a significant increase in dielectric tangent loss and electrical conductivity, a decrease in permittivity, and an increase in the degree of phase transition blurring. The magnetic properties for all P-T composite compositions at RT were preserved and were 0.31 emu/g, 1.60 emu/g, and 4.56 emu/g for P90-T10, P70-T30, P50-T50, respectively. For the *M-H* hysteresis loop at room temperature, the maximum magnetization increased from 1.17 emu/g for (P90-T10) to 15.18 emu/g for (P50-T50), while the coercive field decreased from 271.8 mT for P90-T10 to 9.7 mT for P50-T50. It is also interesting to maintain the high saturation of the *M-H* magnetic hysteresis loop in the composite with the lowest Terfenol-D content (P90-T10). The magnetic properties for all P-T composite compositions at room temperature were preserved and were 0.31 emu/g, 1.60 emu/g, and 4.56 emu/g for P90-T10, P70-T30, and P50-T50, respectively. For the *M-H* hysteresis loop at RT, the maximum magnetization increased from 1.17 emu/g for (P90-T10) to 15.18 emu/g for (P50-T50), while the coercive field decreased from 0.272 T for P90-T10 to 0.001 T for P50-T50. It is also interesting to maintain the high saturation of the *M-H* magnetic hysteresis loop in the composite with the lowest Terfenol-D content (P90-T10). Due to the tendency to combine with oxygen and the high electric conductivity of Terfenol-D, limiting its amount in the composite composition is appropriate. At 10% of Terfenol-D, the composite has good dielectric properties, and the magnetic parameters remain satisfactory.

## 1. Introduction

Natural single-phase materials with multiferroic properties are rare, and their magnetoelectric effect is weak. Moreover, they usually occur at too-low temperatures, limiting the possibility of their wide application [1]. Creating a two-phase composite material containing a ferroelectric and magnetic phase, as proposed by Van Suchtelens [2], initiated intensive research on various types of multiferroic composites. The degree of coupling of electrical and magnetic subsystems in such composites is dependent primarily on the properties of each composite component and their percentage. A valuable feature of composites is the possibility of designing their structure to obtain assumed properties, which considerably broadens the possibilities of applying multiferroic composites compared to the options for the individual composite phases. Multiferroic composite materials obtained in various forms are characterized by much greater magnetoelectric coupling (above room temperature), significantly expanding their application possibilities in various microelectronic devices [1,3]. Nanostructured thin composite layers based on BaTiO_3_ and CoFe_2_O_4_ presented in the work of Zheng [4] are promising. Magnetoelectric materials are used as voltage-current and magnetoelectric transducers, tunable devices (microwave tunable, bandpass filters), energy harvesters, sensors, and transductors (e.g., DC/AC magnetic field, force, current intensity, hydrogen detectors, current intensity), as materials for information recording and reading media, and as materials for medical applications [5,6,7,8,9,10,11,12]. Multiferroic properties can also be used in interference sensors sensitive to field changes, during the precise control of electrical and magnetic fields as well as temperature and pressure, in broadband detectors of the far infrared, and in investigations of the submillimeter electromagnetic spectrum, and as pyroelectric sensors, oscillators, and vibrators [1,2,11,12]. The functional properties of ferroelectric–ferromagnetic composite materials constitute an interesting basis for applications in the field of tuned filter engineering and energy harvesting, e.g., towards the possibility of using them for shielding residential (service) premises against low- and high-frequency electromagnetic radiation while ensuring acceptable electromagnetic compatibility with the environment [13].

Strong ME coupling, with large magnetostriction and high piezoelectric parameters, is critical for developing new types of memory devices and tunable microwave devices [11,12,13,14,15,16,17,18,19,20,21,22,23]. In contrast, magnetoelastic coupling is appropriate for sensors and actuators [24]. The large group of magnetoelectric composites includes materials based on ferrites and piezoelectrics (e.g., PZT, PMN-PT, PFN, BT) [25,26,27,28,29], magnetic alloys/metals (e.g., Terfenol-D, Metglas) and piezoelectrics, and on the combination of Terfenol-D, piezoelectrics, and polymers [30]. The multiferroic properties of composites depend, among other things, on the properties of the individual phases forming the composite, the method of mutual connection, and the ratio of their volumes. According to R.E. Newnham [31], in composite materials, each phase can be connected in three mutually perpendicular directions, in two directions, in one direction, or may not be connected at all. In two-phase ceramic composite materials, the most common types of connecting phases are as follows [32]: 0–3 particulate composite (consists of piezoelectric and magnetic ceramic grains), 2–2 laminate composite (consists of piezoelectric and magnetic layers), and 1–3 fiber/rod composite (consists of fibers of one phase embedded in the matrix of another phase). The effective magnetoelectric coefficient depends mainly on the microstructure of the composite, which is related to the properties of the component phases, the volume concentration of the individual phases, the shape of the grains, and the connection of the individual phases [30].

Terfenol-D (Tb_0.3_Dy_0.7_Fe_2_) is a typical material with giant magnetostriction, which is usually used as a sensing or actuator element in bulk or thin-film form [33,34,35,36]. The material is characterized by the possibility of achieving high magnetostriction strains, and the magnetoelastic properties can be modified due to the combination of magnetization and stress pre-history [37,38,39]. A Terfenol-D rod is often designed to work in particular areas by adjusting the bias magnetic field and the pre-stress. The Terfenol-D rod is very sensitive to temperature, bias, and magnetic field due to the induced eddy current [40,41]. Moreover, the pre-stress and temperature influence the magneto-thermo-mechanical characteristics that vary with the magnetic field’s intensity, affecting the optimal functioning of Terfenol-D-based sensors and actuators [36,42]. Unfortunately, Terfenol-D is also brittle and can only withstand minor strains to failure. It is often combined with a polymer to minimize this feature and form a more beneficial combination [43]. For piezoelectric composites, magnetostrictive Terfenol-D composites can have various connectivity levels [31], and 0–3, 1–3, and 2–2 connectivity are the most common [43]. Studies on various types of composites based on Terfenol-D have shown several properties. For example, in [44], it was shown that increased magnetostriction can be achieved in laminated Terfenol-D/epoxy particulate composites. At the same time, higher values of the magnetoelectric coefficient can be obtained thanks to laminated structures of Terfenol-D plies intersecting with piezoelectric laminas [45].

A promising way to obtain modern and high-performance materials is to produce a variety of physical properties in one material (responsive to various factors—external stimuli) to achieve its high functionality. Obtaining a 0–3 particulate composite based on Terfenol-D and a piezoelectric PZT in the form of a single material will allow, for example, the avoidance of negative phenomena occurring in the boundary areas of layers in Terfenol-D layered composites. In numerous works [46,47,48,49], attempts were made to obtain two-phase composite materials based on ferrites and BaTiO_3_ or PZT-type materials using conventional sintering methods. However, the value of the magnetoelectric coefficient *α*_ME_ was very low. In addition, the studies presented in the work of Ruy [50] showed that the sintering temperature significantly affects the values of *α*_ME_. Strong ME coupling was presented in layered composites consisting of ferrite and ferroelectric materials like relaxors of PZN-PT (lead zinc niobate–lead titanate) and PMN-PT (lead magnesium niobate–lead titanate) [51,52]. Despite many experiments aimed at obtaining a strong magnetoelectric effect (ME) in ceramic composite materials, many technological issues still require solving and refinement. They are concerned with improving the technological process towards achieving the appropriate dispersion of the ferrite phase in the ferroelectric phase matrix, obtaining a high-density composite material, and eliminating interphase diffusion between the two component phases [30]. In the case of layered laminates, the critical factor is to avoid the reaction/diffusion at the interface of two phases and to improve the sintering process of the ferroelectric phase and the magnetic phase with good surface contact. In terms of application, strong electromagnetic coupling between the layers is important. However, ceramic composites with high permittivity values are also highly desirable for resonant (capacitor–coil) applications [30].

Most works on Terfenol-D-based composites concern the study of samples in laminated layered composites [53,54,55,56,57,58,59,60,61]. The inspiration for this work was to obtain a 0–3 particulate composite based on magnetic and ferroelectric materials using powder classical technology. A magnetic material with strong electrostrictive properties (Terfenol-D (T) with a chemical composition of Tb_0.3_Dy_0.7_Fe_2_) and a material with high dielectric properties (doped PZT-type material with a chemical composition of Pb_0.9_Ba_0.1_(Zr_0.53_Ti_0.47_)_0.95_Nb_0.04_O_3_ (P)) were selected for the experiment. Three composite compositions were obtained with the following P/T proportions, 90/10 (P90-T10), 70/30 (P70-T30), and 50/50 (P50-T50), using the pressureless sintering method in an argon environment. In the case of the above materials, the 0–3-type phase connection is difficult to implement, especially using conventional sintering methods. This is mainly due to the difficulty in obtaining a uniform distribution of the magnetic component grains in the ferroelectric component matrix. Since Terfenol-D belongs to strongly oxidizing materials, the assumption of the work was to “embed” the magnetic component in the ferroelectric matrix, preventing the possibility of its connecting with oxygen while maintaining the magnetic properties of Terfenol-D. So far, there have been no literature reports on the studies of 0–3 particulate composites obtained by combining the chemical compositions mentioned above, which is a novelty of this work.

## 2. Materials and Methods

### 2.1. Technology Process

In this work, three compositions of two-phase composites with a phase connection type of 0–3 were obtained based on PZT powder of the composition Pb_0.9_Ba_0.1_(Zr_0.53_Ti_0.47_)_0.95_Nb_0.04_O_3_ (P) and Terfenol-D powder (T) in the P/T ratios 90/10 (P90-T10), 70/30 (P70-T30), and 50/50 (P50-T50). The P ceramic material was obtained with solid-state reaction technology, using a mixture of simple oxides PbO (POCH, Gliwice, Poland, 99.99%), ZrO_2_ (Aldrich, St. Louis, MO, USA, 99.5%), BaCO_3_ (POCH, Gliwice, Poland, 99.99%), Nb_2_O_5_ (Sigma-Aldrich, St. Louis, MO, USA, 99.9%), and TiO_2_ (Merck, Darmstadt, Germany, 99.99%). The ceramic powders were mixed using a wet method for 15 h in a planetary ball mill (Fritsch, Pulverisette 6, Idar-Oberstein, Germany), and synthesis was conducted at 950 °C/8 h. A commercial powder of the composition Tb_0.3_Dy_0.7_Fe_2_ (T), i.e., Terfenol-D (TdVib, LLC., Ames, IA, USA), was used as the magnetic material. The technological process of obtaining composite materials Pb_0.9_Ba_0.1_(Zr_0.53_Ti_0.47_)_0.95_Nb_0.04_O_3_-Tb_0.3_Dy_0.7_Fe_2_ (P-T) was carried out in a protective argon atmosphere. Powders of the P and T materials were mixed in proportions of 90/10, 70/30, and 50/50 for 1 h. Then, the powders were pressed into compacts of 8 mm in diameter under a pressure of 300 MPa and placed in tightly sealed ceramic crucibles in a powder backfill (base material, i.e., P-T powder mixture). Three P-T composite compositions (P90-T10, P70-T30, and P50-T50) were sintered in a muffle furnace using the pressureless method at 1200 °C/12 h in an argon-protective atmosphere.

### 2.2. Measurements Methods

X-ray measurements were performed at room temperature RT on a diffractometer Phillips X’Pert (Panalytical, Eindhoven, The Netherlands) in the 2*θ* angle range from 10° to 70°. The microstructure and EDS chemical analysis (point, linear, and surface analyses) of P-T composites were conducted on the FESEM field emission scanning electron microscope Jeol JSM-7100F TTL LV with energy dispersive spectrometer EDS (Jeol Ltd., Tokyo, Japan). In the case of the microstructure tests, two types of imaging capture were made: the standard SB (connection of signals secondary and backscattered electrons) and the BE (signal from backscattered electrons). The average grain size was estimated in the ImageJ 1.37v program (LOCI, University of Wisconsin-Madison, Madison, WI, USA) based on FESEM microstructure images. Dielectric tests were performed on the QuadTech 1920 Precision LCR meter (QuadTech, Maynard, MA, USA) from RT to 400 °C and at frequencies from 1 kHz to 1 MHz. DC electric conductivity tests were conducted using a digit multimeter Keysight 34465A (Keysight, Santa Rosa, CA, USA) from room temperature to 450 °C. Magnetic properties were obtained by applying the Quantum Design PPMS system (PPMS 7T ACMS module, San Diego, CA, USA) within a temperature range from −263 °C to 30 °C.

## 3. Results and Discussion

### 3.1. Microstructure and EDS Analysis

**Figure 1:**
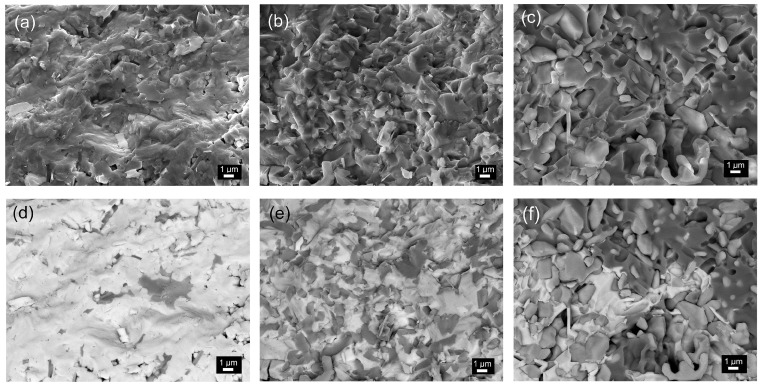
FESEM microstructure images of P-T composites (cross-fracture of the samples) made in SB mode (**a**–**c**) and BE mode (**d**–**f**): (**a**,**d**) P90-T10, (**b**,**e**) P70-T30, and (**c**,**f**) P50-T50.

**Figure 2:**
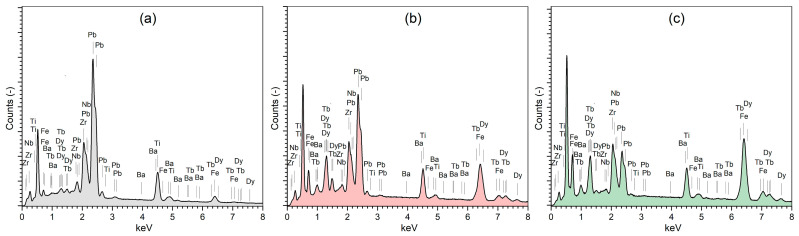
Surface EDS analysis of composite materials P-T: (**a**) P90-T10, (**b**) P70-T30, and (**c**) P50-T50.

The microstructural images of composite materials made on the cross-section of the samples are presented in Figure 1. In the case of the P90-T10 composite, a microstructure with a tightly packed grain of ferroelectric material (constituting the composite matrix) is observed, which surrounds the grains of the magnetic component (Figure 1a). In the case of a higher content of Tefenol-D in the composite, there is an increase in the inhomogeneity of the distribution of the magnetic component grains in the matrix of the P material. At the same time, the internal porosity of the composite materials increases (Figure 1b,c). The surface EDS analysis confirmed the presence of all elements in the composite compositions (Figure 2), which shows a clear increase in iron (Fe), dysprosium (Dy), and terbium (Tb) with the increase in the share of Terfenol-D in the composite. For the P50-T50 composition, the strongest maxima originating from Fe, Tb, and Dy are observed around 6.4 keV and above 7 keV, while the weakest maxima in this region occur for the P90-T10 composition. In contrast, the strongest maxima originating from Pb (around 2.36 keV) and Zr (around 2.06 keV) occur for the P90-T10 composition, and the weakest maxima in these regions occur for the P50-T50 composition, respectively. A detailed EDS analysis also revealed the decomposition of two main components of the composite material. Due to the strong oxidizing properties of Terfenol-D, as well as the tendency towards lead escape under sintering at high temperatures, dysprosium, terbium, and iron combine with the elements of the ferroelectric component P, creating new phases. In the microstructural SEM images made using the BE technique, this is visible in the form of areas of different shades (Figure 1d,e).

**Figure 3:**
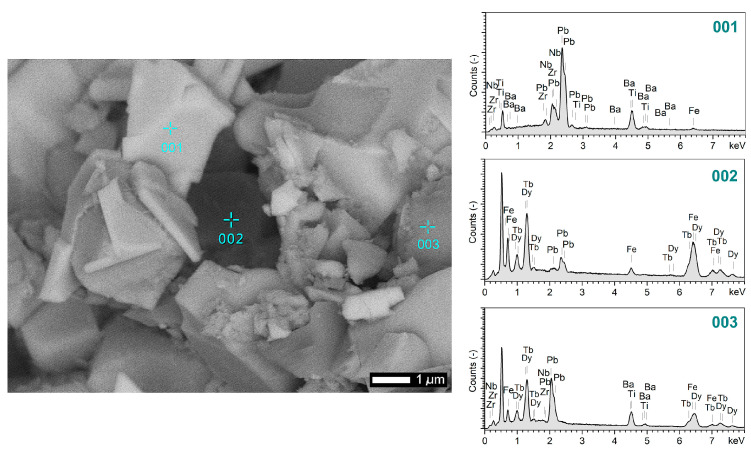
Point EDS analysis for composite P50-T50.

Based on the point EDS analysis (Figure 3) performed for the P50-T50 composite, it was shown that the bright areas (001) are dominated by the presence of lead (Pb), titanium (Ti), zirconium (Zr), and barium (Ba), with a slight presence of iron (Fe). The dark areas (002) are rich in dysprosium (Dy), terbium (Tb), and iron (Fe), with a small presence of lead (Pb). In turn, the areas with a gray shade (003) correspond to regions rich in dysprosium (Dy), terbium (Tb), and zirconium (Zr), with the presence of lead (Pb), titanium (Ti), and iron (Fe). In the case of the P70-T30 and P90/10 composites, the EDS point analysis showed that the bright areas are rich in Pb and Ti, Zr, and Ba with a small amount of Fe, while the dark areas are rich in Fe and Tb and Dy, with the presence of a small amount of Pb and Zr. EDS analysis confirmed the decomposition of two composite components (P and T), which resulted in additional foreign phases containing magnetic and electric elements. The three areas mentioned above are clearly visible for the compositions with the highest Terfenol-D content, i.e., P50-T50 (Figure 1f). 

**Figure 4:**
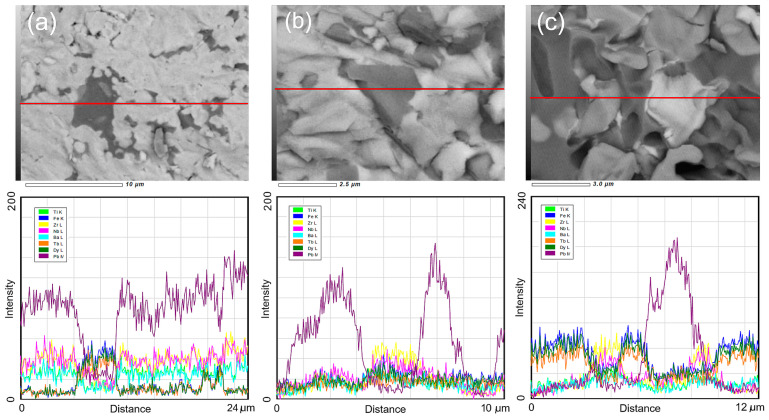
Linear EDS analysis of composite materials P-T: (**a**) P90-T10, (**b**) P70-T30, and (**c**) P50-T50.

The occurring decomposition phenomenon in P-T composite materials is also confirmed by the linear EDS analyses of the chemical composition in the light and dark areas of the microstructure (Figure 4). In the case of sample P50-T50, the linear analysis was carried out through three areas of the microstructure: light, gray, and dark. In the light area, a high intensity of the elements Pb, Zr, and Ti is observed; in the dark area, Dy, Tb, and Fe; while in the gray area, an increased share of Dy, Tb, Zr, Ti, Fe, and a lower share of Pb were recorded.

### 3.2. Crystal Structure

**Figure 5:**
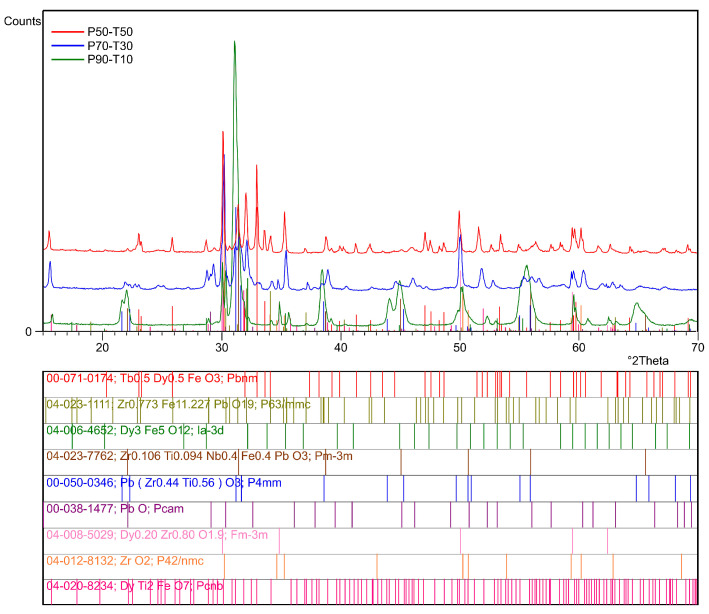
XRD patterns of the P-T composites at room temperature.

Figure 5 compares room temperature X-ray patterns from P90-T10, P70-T30, and P50-T50 samples. Phase analysis showed that in addition to the main phase of the composite, i.e., the type-PZT material (00-033-0784 pattern with tetragonal crystal structure), X-ray analysis revealed an occurring decomposition of the base composition of the P-T composites, which occurs for all compositions with varying intensity, depending on the relative amount of each composite component. It was found that the more Terfenol-D is present in the composite composition, the more intensified the composition decomposition process becomes.

For the P90-T10 composition, a partial overlapping of reflections belonging to the following phases was identified: Zr_0.773_Fe_11.227_PbO_19_ (ICDD PDF5+ 04-023-1111), Zr_0.106_Ti_0.094_Nb_0.4_Fe_0.4_PbO_3_ (ICDD PDF5+ 04-023-7762), Dy_0.2_Zr_0.8_O_1.9_ (ICDD PDF5+ 04-008-5029), and PbZr_0.44_Ti_0.56_O_3_ (ICDD PDF5+ 00-050-0346). In the case of the phase analysis of the P70-T30 composite material, the phase analysis showed the presence of the following phases: Zr_0.773_Fe_11.227_PbO_19_ (ICDD PDF5+ 04-023-1111), Zr_0.106_Ti_0.094_Nb_0.4_Fe_0.4_PbO_3_ (ICDD PDF5+ 04-023-7762), Dy_0.2_Zr_0.8_O_1.9_ (ICDD PDF5+ 04-008-5029), PbZr_0.44_Ti_0.56_O_3_ (ICDD PDF5+ 00-050-0346), Tb_0.5_Dy_0.5_FeO_3_ (ICDD PDF5+ 00-071-0174), DyTi_2_FeO_7_ (ICDD PDF5+ 04-020-8234), ZrO_2_ (ICDD PDF5+ 04-012-8132), PbO (ICDD PDF5+ 00-038-1477), and Zr_0.25_Ti_0.75_PbO_3_ (ICDD PDF5+ 04-011-7313). For the equilibrium proportion of P50-T50 composite components, X-ray phase analysis revealed the presence of Zr_0.106_Ti_0.094_Nb_0.4_Fe_0.4_PbO_3_ (ICDD PDF5+ 04-023-7762), Zr_0.773_Fe_11.227_PbO_19_ (ICDD PDF5+ 04-023-1111), Dy_0.2_Zr_0.8_O_1.9_ (ICDD PDF5+ 04-008-5029), PbZr_0.44_Ti_0.56_O_3_ (ICDD PDF5+ 00-050-0346), Tb_0.5_Dy_0.5_FeO_3_ (ICDD PDF5+ 00-071-0174), DyTi_2_FeO_7_ (ICDD PDF5+ 04-020-8234), and Zr_0.25_Ti_0.75_PbO_3_ (ICDD PDF5+ 04-011-7313) phases. The reason for the composition decomposition phenomenon is the strong tendency of Terfenol-D to combine with oxygen, which results in the relatively easy combination of terbium, dysprosium, and iron with the elements of the ferroelectric material, creating new foreign phases. An additional element intensifying this process may be a temperature increase that is too rapid during sintering in the technological process.

### 3.3. Dielectric Properties

**Figure 6:**
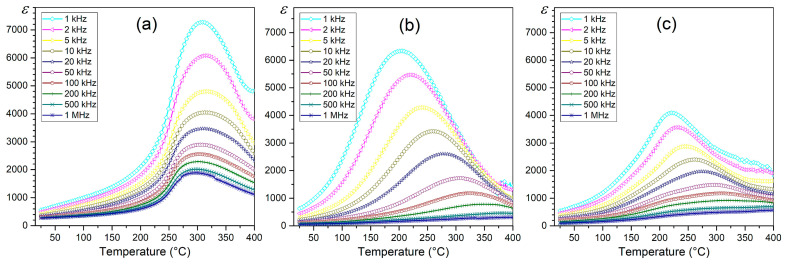
Permittivity vs. temperature of P-T composites: (**a**) P90-T10, (**b**) P70-T30, and (**c**) P50-T50.

**Figure 7:**
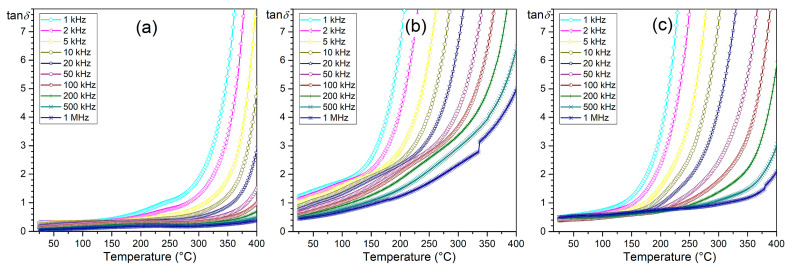
Dielectric loss factor (tan*δ*) vs. temperature of P-T composites: (**a**) P90-T10, (**b**) P70-T30, and (**c**) P50-T50.

**Figure 8:**
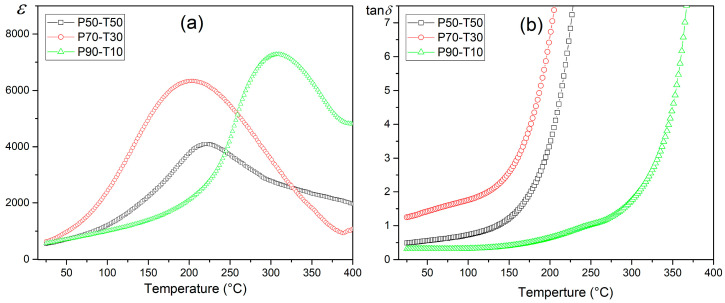
Permittivity (**a**) and dielectric loss factor (tan*δ*) (**b**) vs. temperature of P-T composites for 1 kHz.

The influence of the amount of Terfenol-D in the P-T composite on its dielectric properties is also visible in the temperature courses of *ε*(*T*) (Figure 6) and tan*δ*(*T*)—Figure 7. Dielectric studies have shown that high permittivity values in composite materials are maintained. However, with the increase in the amount of Terfenol-D, the ferroelectric/parelectric phase transition is strongly blurred, and the temperature region of the phase transition is widened (Figure 8a). This indicates a reduced degree of ion ordering in the B position of the compound, among other things, caused by the formation of additional phases, which is confirmed by the previously conducted XRD and EDS studies. The composition P90-T10 with high permittivity values P90-T10 *ε* = 570 at RT and *ε*_m_ = 7300 at *T*_m_ (1 kHz) maintains the highest stability of dielectric parameters.

The P90-T10 composite also has the lowest dielectric tangent loss values (for 1 kHz, tan*δ* = 0.32 for RT and tan*δ* = 1.94 for *T*_m_). For the composition with the highest amount of Terfenol-D (P50-T50), the permittivity values decrease significantly, and the increase in dielectric tangent loss values is significant in the entire measurement range for P70-T30 and P50-T50 compositions (Figure 7b and Figure 8b). At RT for P70-T30 and P50-T50, tan*δ* is 1.24 and 0.45, respectively. A lack of the expected trend of increasing tan*δ* with the increase in the amount of Terfenol-D was also observed. The highest tan*δ* values were observed for the composition 70/30, which deviated from this regularity. The lack of this regularity was also confirmed by the direct current electrical conductivity studies, which will be discussed later in the paper.

The paper [62] presents the dielectric tests of multiferroic composites based on PZT-type material and zinc–nickel ferrite (P-F) obtained by different sintering methods. Dielectric studies showed that the composites obtained with ferrite exhibit significant high-temperature dielectric dispersion, which powerfully blurs the phase transition, especially at lower frequencies [28,62]. In the case of the P90-T10 composite, such a phenomenon does not occur, and the permittivity values are higher than those of the P-F ferrite composite obtained by the pressureless method [62]. However, the P90-T10 composite exhibits higher dielectric tangent loss. Similar trends concerning phase transition blurring were observed by the authors of [63], who studied PZT–nickel ferrite (PZT-NZF) and PZT–cobalt ferrite (PZT-CF) composites. In the case of PZT-CF, the permittivity values were at a similar level as in the P90-T10 composite, while in the case of PZT-NZF (for the introduced ferrite of the composition Ni_0.7_Zn_0.3_Fe_2_O_4_), two-times-lower permittivity values were obtained. Also, the dielectric loss values of PZT-NZF and PZT-CF composites presented in [63] are higher than those obtained for the comparable composition P90-T10.

### 3.4. DC Electric Conductivity

**Figure 9:**
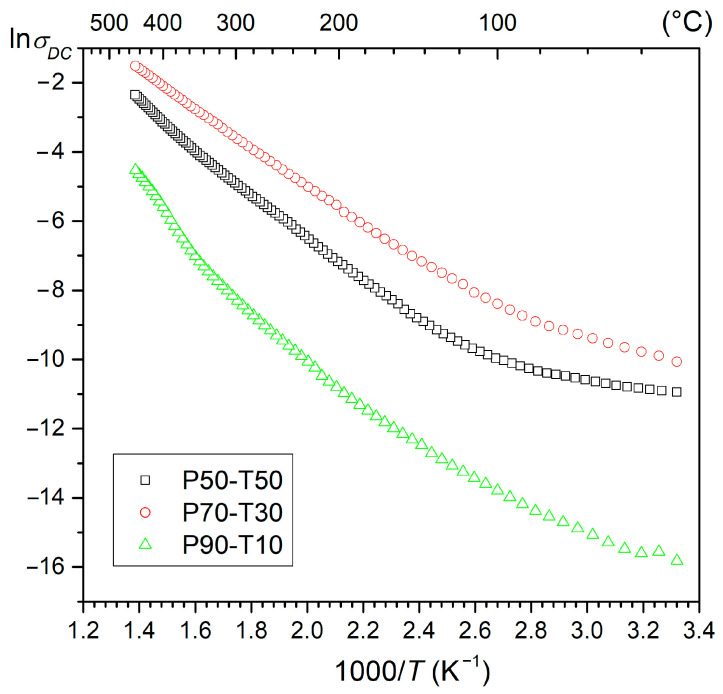
The ln*σ*_DC_(1000/*T*) relationship for P-T composites.

The DC electrical conductivity tests of P-T composite materials are presented in Figure 9. The tests confirmed that Terfenol-D significantly reduces the resistance of composite materials by increasing the electrical conductivity. The lowest electrical conductivity is shown by the P90-T10 composition, i.e., the composite with the highest P component content, while the P70-T30 composition shows the highest conductivity. At room temperature, the resistivity values are 7.4 × 10^6^ Ωm, 2.3 × 10^4^ Ωm, and 5.4 × 10^4^ Ωm for P90-T10, P70-T30, and P50-T50, respectively. With increasing temperature, there is a systematic increase in electrical conductivity; above 110 °C, this increase is more rapid. The lack of the expected trend of linear increase in electrical conductivity with increasing Terfenol-D content in composite compositions containing the highest amounts of Terfenol-D (Figure 9) may be related to the different degree of composition decomposition and formation of new phases, as well as increased precipitation of pollutants at the phase boundary.

### 3.5. Magnetic Properties

**Figure 10:**
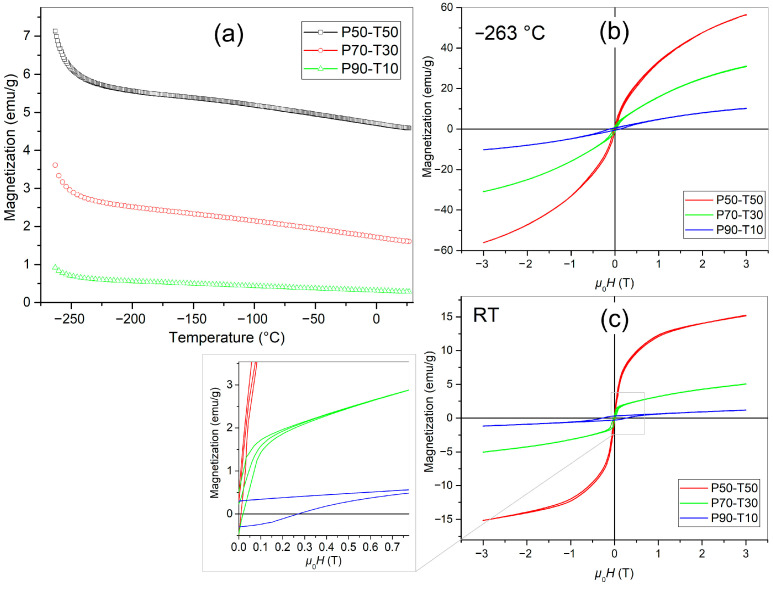
Temperature dependencies of magnetization (**a**) and magnetic hysteresis loops at −262 °C (**b**) and at RT (**c**) for P-T multiferroic ceramic composites.

The magnetic properties studies for P-T composite materials are presented in Figure 10. At −263 °C, the magnetization values are 0.95 emu/g, 3.64 emu/g, and 7.13 emu/g for P90-T10, P70-T30, and P50-T50 composite samples, respectively. Increasing the amount of Terfenol-D increases the magnetic response of the P-T composite material. The *M-H* hysteresis loops at −263 °C (Figure 10b) and RT (Figure 10c) are slender and narrow. After increasing temperature up to about –248 °C, there is a rapid decrease in magnetization. Above −248 °C, the decrease in magnetization is not as rapid but occurs in a monotonic manner (see Figure 10a). This dependence results from a superposition of the two magnetic components attributed to paramagnetic and ferro/ferri magnetic phases. At RT, for all P-T composite compositions, the magnetic properties are preserved, and the magnetizations are 0.31 emu/g, 1.60 emu/g, and 4.56 emu/g for P90-T10, P70-T30, and P50-T50 samples, respectively. With the increase in the number of magnetic components, the magnetic hysteresis loops *M-H* (Figure 10b,c) show an increase in the magnetization value, but the coercive field value decreases. This is clearly visible in Figure 10c for the RT, where the maximum magnetization increases from 1.17 emu/g for (P90-T10) to 15.18 emu/g for (P50-T50), while the coercive field value decreases from 0.272 T for P90-T10 to 0.001 T for P50-T50. Comparing the results of magnetic tests of the P90-T10 composite with the P-F ferrite composite obtained in [62], one can observe a different nature of the magnetic hysteresis loop, with a wide coercive field but a lower magnetization value.

Comparing the magnetic characteristics with the phase composition determined by XRD measurement, it is evident that the applied technology caused the decomposition of the Terfenol-D component into rare earth oxides. Moreover, iron also formed some oxides. The magnetic hysteresis loop measured at room temperature revealed the dominance of ferro/ferrimagnetic phases, which can be attributed to iron in oxidation states +2 and +3. On the other hand, the paramagnetic response plays the main role at lower temperatures. Such behavior is undoubtedly related to rare earth oxides, which by nature exhibit paramagnetic properties.

## 4. Conclusions

The experiment and experimental studies have shown that obtaining a 0–3 particulate composite based on a PZT-type ferroelectric material and magnetic Terfenol-D using the classic sintering method with good magnetic and dielectric properties in one material is possible. However, chemical composition studies and X-ray studies have revealed the occurrence of decomposition of composite compositions, which results in new phases, most of which contain rare earth elements. Despite the occurrence of composition decomposition, the obtained composite materials retain both dielectric and magnetic properties at room temperature. Also fascinating is the high coercive field and the maintenance of high saturation of the magnetic hysteresis loop in the composite with the lowest Terfenol-D content (P90-T10). Since Terfenol-D has high conductivity and the tendency to combine with oxygen, limiting its amount in the composite composition is advisable (at 10%, the magnetic properties are maintained at a satisfactory level). It is necessary to optimize the technological process parameters further to obtain the optimal properties of the 0–3 particulate composite based on Terfenol-D and PZT-type material. This concerns not only the preservation of the elemental compositions of the composite, but also the obtaining of high homogeneity of the microstructure of the composite material with uniform distribution of the magnetic grains of Terfenol-D “embedded” in the ferroelectric matrix. This is required to obtain a high magnetoelectric coefficient of the composite material. Such a material may be an alternative to layered composites in the future, avoiding negative phenomena occurring in layered boundary areas. Further experiments will be conducted in this direction using an alternative sintering atmosphere, e.g., Al_2_O_3_ backfill.

## Data Availability

Data are contained within the article.
